# Birt-Hogg-Dubé Syndrome Caused by a Novel Mutation in the FLCL Gene

**DOI:** 10.1155/2018/4173704

**Published:** 2018-11-07

**Authors:** Charles Volk, Gregory Matwiyoff

**Affiliations:** Medical Corps, United States Navy, Pulmonary Department, Naval Medical Center, San Diego, 34800 Bob Wilson Drive, San Diego, CA 92134, USA

## Abstract

**Background:**

Birt-Hogg-Dubé syndrome is a genetic disorder characterized by skin fibrofolliculomas, cystic lung disease, and bilateral renal tumors. It has also been implicated in the formation of tumors in other organs, particularly thyroid and colon. This case presents a young female presenting with only cystic lung disease and kidney tumors, identified as having a never before identified heterozygous mutation in the folliculin (*FLCN*) gene which is the likely cause of her syndrome.

**Case Presentation:**

A 34-year-old female was found to have bilateral renal masses, 2.4 cm on the right and 7.6 cm on the left, as well as multiple, small cysts in the lungs. Chest imaging further characterized the lung cysts as being basilar predominant with the largest measuring 1.6cm. The left kidney mass was resected with a partial nephrectomy with final pathologic diagnosis of an oncocytoma. Genetic testing was undertaken as she did not have characteristic skin findings. A previously undescribed mutation in the* FLCN* gene (c.780-2A>G) was identified with no matches in the human genetic mutation database (HGMD). Review of that database identified over 160 separate mutations in the* FLCN* gene. Extensive history did not identify any family members who had similar disease processes suggesting that this could be a spontaneous mutation in the proband.

**Conclusions:**

This case highlights that the traditional view of Birt-Hogg-Dubé syndrome as having a strong familial component may be incorrect and that spontaneous mutation may be more common than previously thought. Also notable is the fact that this patient had no characteristically described fibrofolliculomas that traditionally are the hallmark of the condition. This case suggests that genetic testing should be obtained in all suspected cases of Birt-Hogg-Dubé syndrome as the patient may not present with the typical skin findings and may also present with no family history consistent with this disorder.

## 1. Background

Birt-Hogg-Dubé syndrome is marked by kidney tumors, cystic lung disease, and skin fibrofolliculomas. It has also been implicated in the formation of tumors in other organs, particularly thyroid and colon though these have had inconsistent reports in the literature [1, 2]. There are isolated case reports of other multiple tumors forming in patients with Birt-Hogg-Dubé syndrome, though these have not thus far shown a clear association with the mutation of the folliculin (*FLCN*) gene responsible for the syndrome. It traditionally presents in a familial, autosomal dominant pattern with high penetrance, but there are few reports in the literature of unique mutations presenting with this syndrome [3, 4].

Patients with this condition are at greatly increased risk of kidney cancer and pneumothorax compared to the general population. Kidney tumors can range from benign to malignant, though their malignancies tend to be of lower grade. However, their risk of kidney cancer is roughly seven times that of the general population. Pneumothorax is a complication in roughly 30% of people with Birt-Hogg-Dubé syndrome and is secondary to lung cysts. However, lung dysfunction is usually minimal with most people showing normal pulmonary function testing or minimal airway obstruction [5]. The skin findings are typically fibrofolliculomas, usually on the face, and are the most common finding in Birt-Hogg-Dubé syndrome. These skin lesions may be mildly disfiguring but are usually benign.

This genetic, autosomal dominant condition is caused by a mutation of the* FLCN* gene on chromosome 17. Its exact function is unknown, but it is thought to be a tumor suppressor gene. Historically, this was thought to be a primarily familial disease, but reports like the one below identify isolated cases identifying a novel FLCN mutation.

## 2. Case Report

**Figure 1:**
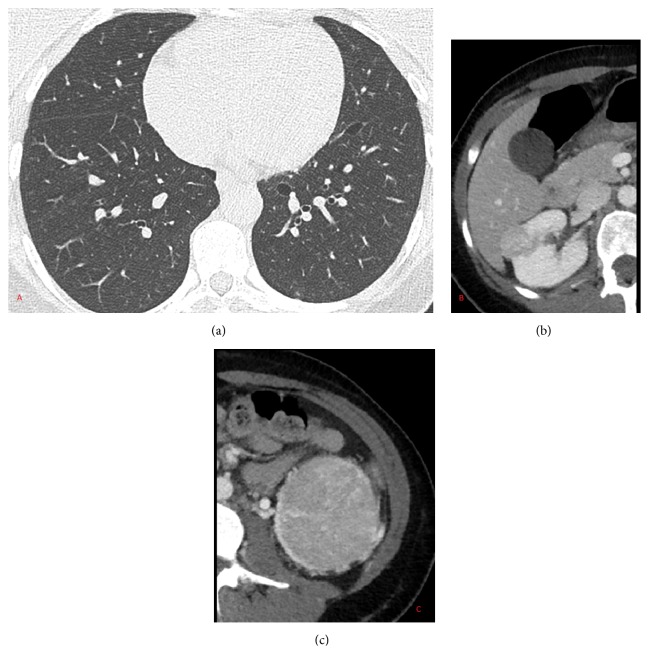
Image showing lung cysts (a), right kidney mass of 2.4 cm (b), and left kidney mass of 7.6 cm (c), which was later identified as an oncocytoma.

A 34-year-old female was seen in the emergency department for abdominal pain. Her workup included a CT abdomen where she was found to have bilateral renal masses: 2.4cm on the right and 7.6 cm on the left (Figure 1). Also noted were multiple small cysts in the lung bases. The rest of the workup was unremarkable and her abdominal pain resolved with conservative management alone. She was referred to urology where it was recommended that her left kidney tumor be resected and to defer the right pending pathology results. Notably, she had no prior medical history and no relevant surgical history and had otherwise been healthy and well. She has two siblings without lung, skin, or kidney symptoms and her parents are likewise healthy. She has a 15-year-old son who is healthy. There were no consistent skin findings on exam.

Pulmonary evaluation with a CT of chest identified basilar predominant multiple lung cysts with the largest cysts measuring approximately 1.6cm. Several <6mm partially solid nodules were noted as well. Spirometry, diffusion capacity, and plethysmography were all within normal limits. She did complain of mild dyspnea, but that this was intermittent and had a significant anxiety component.

She eventually underwent resection of the left kidney mass with a partial nephrectomy and a final pathologic diagnosis of an oncocytoma, which is a typical tumor type for Birt-Hogg-Dubé syndrome. Her postoperative course was unremarkable with a planned sequential right nephrectomy pending further evaluation.

Birt-Hogg-Dubé syndrome was suspected given this patient's basilar predominant multiple lung cysts and bilateral renal masses, but without skin findings the diagnosis was in question. There are no universally accepted diagnostic criteria, but typically either skin findings or a pathologic mutation must accompany the lung and kidney pathology to solidify the diagnosis. Genetic testing was thus obtained via a blood sample. The* FLCN *gene of the patient was sequenced with any deletions or duplications included. It showed a heterozygous mutation of the* FLCN* gene (c.780-2A>G) predicted to disrupt the canonical splicing acceptor site of exon 8 of* FLCN.* This mutation has not been previously described in the human genetic mutation database (HGMD). On further review, the HGMD has over 160 individual mutations identified in the* FLCN* gene. However, the majority of identified familiar mutations in the* FLCN* gene are confined to two separate mutations (c.1285dupC and c.1285delC).

The consistent* FLCN* gene mutation along with renal and lung findings supported the diagnosis of Birt-Hogg-Dubé syndrome. The patient was referred to a medical geneticist. It was noted that the patient was the child of second cousins but otherwise she had no family with syndromic complaints consistent with Birt-Hogg-Dubé syndrome. However, clinical manifestations of this disease process can be subtle and given that her kidney and lung manifestations were found incidentally, these could have remained unidentified in her family members. Genetic testing of her family is pending.

On follow-up imaging, her lung cysts have remained stable, but her right kidney tumor has increased to 2.8cm (previously 2.4cm) and a new, small 8mm satellite lesion was identified. Based on these findings resection was recommended for her given the high rate for malignant transformation.

## 3. Discussion

This case presents a novel* FLCN* mutation causing the lung and kidney manifestations of Birt-Hogg-Dubé syndrome but none of the more typical skin findings. Given no consistent complaints from her family history, this may be a spontaneous mutation in the proband. However, given the purely visceral organ effects of this particular mutation, other family members may have been affected subclinically. The number of individual* FLCN* mutations (>160) in the HGMD also suggests that novel mutation in the* FLCL* gene may be a more common cause of Birt-Hogg-Dubé syndrome than has previously been described.

This case is particularly notable for her mutation causing only lung and kidney findings while sparing the integument. It is possible that the plethora of novel mutations of* FLCN* belies a much more variable clinical presentation than previously thought.

We recommend genetic testing in all cases of suspected Birt-Hogg-Dubé syndrome even without family history as spontaneous mutations may be a common cause of this disorder. With more widespread availability of genetic analysis in patients and their family members, the true rate of spontaneous mutation will hopefully be elucidated and future studies on Birt-Hogg-Dubé syndrome are warranted to identify the true novel mutation rate as well as genetic factors that could predict organ systems affected.
